# Perceptions of the nursing profession among first-year health profession students: A cross-sectional study in Palestine

**DOI:** 10.1371/journal.pone.0334933

**Published:** 2025-10-24

**Authors:** Kareem Sbaih, Mohammad Qtait, Nesreen Alqaissi, Fuad Farajalla

**Affiliations:** Nursing College, Palestine Polytechnic University, Hebron, Palestine; Ajman University, UNITED ARAB EMIRATES

## Abstract

**Background:**

Nursing plays a vital role in healthcare systems worldwide, yet in many societies including Palestine it remains undervalued and misunderstood. First-year health profession students’ perceptions of nursing influence academic engagement, career preferences, and interprofessional relationships. Recent Palestinian studies have shown that resilience, coping strategies, and exposure to clinical environments strongly affect how students view nursing as a profession.

**Objective:**

To assess perceptions of the nursing profession among first-year health profession students at University, and explore the influence of sociodemographic characteristics and prior healthcare exposure.

**Methods:**

A descriptive cross-sectional study was conducted among 214 first-year students in health-related programs between 10 March and 10 April 2025. Data were collected using a culturally adapted 26-item Nursing Image Scale (NIS). Descriptive statistics, t-tests, one-way ANOVA, multivariable regression, and ANCOVA were applied, with significance set at p < 0.05.

**Results:**

The mean perception score was 3.99 out of 5 (79.8%), reflecting moderately positive attitudes. Nursing students reported slightly higher scores (80%) than non-nursing peers (78.4%). No significant differences were found by gender, residence, or family ties to nursing. However, prior hospital exposure was significantly associated with more favorable perceptions (p = 0.04). Regression analysis confirmed that gender, age, GPA, and prior clinical exposure predicted higher perception scores.

**Conclusion:**

While overall perceptions of nursing were moderately positive, direct hospital exposure and academic achievement were linked to more favorable views. These findings suggest that integrating structured early clinical experiences and resilience-building strategies into curricula could strengthen the image of nursing in Palestine.

**Limitations:**

The single-institution setting and use of convenience sampling limit generalizability, though findings remain valuable for regional nursing education policy.

## Introduction

Nursing is an essential pillar of healthcare systems, grounded in scientific knowledge, technical competence, and humanistic values. It is a profession that demands critical thinking, compassion, ethical awareness, and lifelong learning. Globally, the perceptions of nursing among students entering health-related programs significantly influence their career preferences, academic engagement, and long-term retention within the health workforce. This is particularly important in Palestine, where the healthcare system continues to face workforce shortages and systemic challenges that could be alleviated by promoting nursing as a viable and respected career path.

The early perceptions of nursing among students in health sciences are shaped by multiple factors, including family influence, media portrayal, gender roles, cultural attitudes, and academic experiences [1]. These perceptions may either attract or deter students from considering nursing as a career option. Misconceptions about nurses as subordinate to physicians or limited to basic care tasks remain prevalent in many societies, undermining the profession’s development and affecting recruitment efforts. In addition, nursing is still viewed as a female-dominated career with limited autonomy and career advancement opportunities, which discourages male participation and influences professional perceptions in academic and social contexts [1–3].

International studies demonstrate that cultural and social constructs strongly shape students’ views. For example, a study among international students reported generally positive perceptions, with gender and motivation for choosing nursing influencing attitudes [4]. Similar findings were documented in Turkey and the United States, where cultural expectations and social narratives determined students’ professional perceptions [5,6]. In contrast, studies from Jordan, Saudi Arabia, and sub-Saharan Africa show that nursing is often regarded as a lower-status profession compared to medicine or pharmacy, which creates barriers to recruitment [7,8].

In the Palestinian context, perceptions of nursing are influenced by both social norms and the broader political environment. Recent studies highlight the psychological and educational challenges faced by Palestinian nursing and allied health students. For example, progressive muscle relaxation significantly reduced anxiety among nursing students during their initial clinical training [9–11]. Stressors and coping strategies among physical therapy students were also documented [12–15]. More recently, resilience and psychological well-being were found to be closely related among Palestinian nursing students [16], while resilience was shown to be a protective factor during political violence in clinical training [17]. These findings suggest that resilience, coping strategies, and early exposure to clinical environments are key determinants of how nursing is perceived among students in Palestine.

The World Health Organization (WHO) and the International Council of Nurses (ICN) have emphasized that strengthening the perceptions of nursing and developing educational pathways are critical strategies for addressing the global nursing shortage [9–11,18]. Educational institutions are therefore urged to identify and address misconceptions early in students’ academic journeys. At University, first-year students from diverse health programs—including nursing, medicine, laboratory sciences, and allied health—often enter with limited knowledge about the scope of nursing. Their perceptions are shaped by media, family influences, and occasional hospital exposure. Understanding these perceptions is essential to inform targeted interventions, foster interprofessional respect, and promote nursing as a respected and autonomous profession within Palestinian society.

Against this background, the present study investigates the perceptions of the nursing profession among first-year health profession students at university By identifying factors that shape students’ attitudes toward nursing, this research aims to inform nursing education, guide policy, and contribute to regional and international discussions on strengthening the nursing workforce.

## Methods

### Research design

This study employed a quantitative, descriptive cross-sectional design to assess the perceptions of the nursing profession among first-year health profession students at University in Hebron, Palestine.

### Population and sampling

The target population consisted of all first-year students enrolled in health profession programs at university during the 2024–2025 academic year. The required sample size was calculated using the Raosoft Sample Size Calculator with a 95% confidence level, 5% margin of error, and a population of 400 students, resulting in a minimum sample size of 197. A total of 214 students were recruited between 10 March and 10 April 2025.

A convenience sampling method was adopted due to accessibility of students during scheduled lectures and practical sessions. This facilitated timely recruitment and ensured the sample exceeded the minimum requirement. While convenience sampling was feasible and efficient, it introduces selection bias and limits generalizability beyond the study setting. These issues are acknowledged as key methodological limitations.

### Inclusion and exclusion criteria

Eligible participants were first-year students enrolled in health profession programs at PPU who provided informed consent. Exclusion criteria included students in non-health faculties, those in second year or higher and bridging students with prior healthcare education or clinical training, as their perceptions may differ. Incomplete questionnaires were excluded from analysis.

### Instrumentation: Cultural Adaptation of the Nursing Perceptions Scale

Perceptions of nursing were measured using a culturally adapted version of the Nursing Perceptions Scale. Because the original instrument was developed in a different cultural and linguistic context, a rigorous adaptation process was conducted according to international guidelines for cross-cultural validation.

The process included forward translation, reconciliation, back-translation, expert panel review, and pilot testing with a sample of first-year health profession students. During adaptation, two items from the original 28-item scale were removed based on three criteria: (1) cultural irrelevance in the Palestinian context, (2) persistent clarity issues noted in cognitive interviews, and (3) poor psychometric performance during pilot testing at PPU. The final Arabic version comprised 26 items across three domains (General Appearance, Communication, and Occupational/Educational Characteristics). Reliability analysis in the main study showed excellent internal consistency (Cronbach’s α = 0.93).

### Pilot study

Prior to the main study, a pilot test was conducted with a sample of 35 from students at to assess the clarity, cultural appropriateness, and psychometric properties of the adapted questionnaire. Feedback from participants indicated that the items were clear and understandable, with no major difficulties in comprehension. The pilot data demonstrated good internal consistency, with a Cronbach’s alpha coefficient of **0.93**, supporting the reliability of the instrument in the Palestinian context.

To avoid contamination and ensure independence of the main dataset, all pilot participants were excluded from the final study sample.

### Data collection

Data collection was conducted in coordination with faculty schedules to minimize disruption. Questionnaires were administered in person, with clear instructions provided to ensure accuracy. Participation was voluntary and anonymous, with students given the option to withdraw at any time.

### Data analysis

Data were analyzed using IBM SPSS Statistics version 29. Descriptive statistics (means, standard deviations, frequencies, and percentages) were used to summarize sociodemographic characteristics and perceptions of nursing.

Independent-samples *t*-tests and one-way ANOVA were applied to examine differences in perception scores across demographic subgroups. To strengthen analytical rigor, multivariable linear regression was conducted with the total NIS score as the dependent variable and key predictors (gender, age, GPA, high school stream, prior hospital exposure, current major, and family member in nursing). Model assumptions were verified, and results were reported as unstandardized coefficients with 95% confidence intervals.

Additionally, Analysis of Covariance (ANCOVA) was used to compare adjusted mean perception scores across groups (nursing vs. non-nursing majors, hospital exposure vs. no exposure) while controlling for confounders. Effect sizes were reported using Hedges’ g and partial η². A p-value < 0.05 was considered statistically significant.

### Ethical consideration

Ethical approval for the study was obtained from the Ethical Committee of the College of Nursing at Palestine Polytechnic University (Approval No. EA/2025/50). All participants provided written informed consent after being fully informed about the study’s objectives, procedures, potential risks, benefits, and their rights, including the voluntary nature of participation and the option to withdraw at any time without consequences. Participation was anonymous, and confidentiality of responses was strictly maintained.

As all participants were university students aged 18 years or older, no minors were involved, and therefore no parental or guardian consent was required. The study adhered to the ethical principles outlined in the Declaration of Helsinki regarding human subject research.

## Results

A total of 214 health profession students participated in the study. The mean age of participants was 18.6 years (SD = 1.19). The majority were female (71.5%, *n* = 153), while males constituted 28.5% (*n* = 61). Most participants resided in villages (53.7%), followed by those living in cities (43.9%) and camps (2.3%). Regarding high school stream, 75.7% (*n* = 162) came from the scientific stream and 24.3% (*n* = 52) from the literary stream. Concerning academic performance, 63.1% had a high school average between 70–79.9, 30.4% between 80–89.9, and 6.5% between 90–99. More than half of the participants were nursing students (56.5%, *n* = 121), while the remaining 43.5% (*n* = 93) were students from other medical and health sciences. A majority (88.8%, *n* = 190) had previous admission to a hospital or accompanied someone during a hospital stay, and 33.6% (*n* = 72) reported having a family member who is a nurse or nursing student. Table 1.

**Table 1:** Socio-demographic characteristics of the participating nurses (N = 214).

Variable	n	%
**Gender**		
Female	153	71.5
Male	61	28.5
**Place of residence**		
City	94	43.9
Village	115	53.7
Camp	5	2.3
**High school stream (Tawjihi)**		
Scientific	162	75.7
Literary	52	24.3
**High school average**		
70–79.9	135	63.1
80–89.9	65	30.4
90–99	14	6.5
**Current major**		
Nursing students	121	56.5
Non-nursing health profession students	93	43.5
**Ever admitted to or accompanied someone in a hospital stay**		
Yes	190	88.8
No	24	11.2
**Having a family member who is a nurse or nursing student**		
Yes	72	33.6
No	142	66.4

*Note. N/n* = Number; % = Percentage.

The study found that the health profession students’ overall perception mean score was 3.99 (SD = 0.60), which corresponds to a transformed score of 79.8%, indicating a moderately positive attitudes based on Bloom’s cut-off scores (Bloom, 1956). Comparing the two groups, the nursing students’ mean score was very slightly higher (M = 4.0, SD = 0.72; 80%), indicating a positive perception, whereas the non-nursing health profession students had a moderately positive attitudes (M = 3.92, SD = 0.37; 78.4%). Table 2.

**Table 2:** Overall perception of nursing image among nursing and non-nursing students using the 26-item Nursing Image Scale (N = 214).

Group	Level of perception	Transformed score (100%)*	M (SD)	Frequency (n)
Nursing students	Positive perception	80.0	4.00 (0.72)	121
Non-nursing health profession students	Moderately positive attitudes	78.4	3.92 (0.37)	93
**Total**	Moderately positive attitudes	79.8	3.99 (0.60)	214

*Note. M* = Mean; SD = Standard Deviation.

*Transformed Score (100%) = (Mean/ 5) × 100*.

Independent samples t-tests and one-way ANOVA were used to test for differences in overall perception mean scores by chosen sociodemographic factors. There were no significant differences found by gender (p = 0.63), stream in high school (p = 0.62), high school average (p = 0.76), current major (p = 0.14), residence (p = 0.82), or family member in nursing (p = 0.38). However, a significant difference was found based on personal hospitalization experience (*p* = 0.04), where participants who have been admitted to a hospital or accompanied someone during a hospital stay had a higher perception score (M = 4.0, SD = 0.58) compared to those without (M = 3.7, SD = 0.71). This suggests that direct or indirect exposure to clinical settings may positively influence perceptions of the nursing profession. Table 3.

**Table 3:** Differences in overall perception mean scores according to selected sociodemographic characteristics (N = 214).

Variable	Group	Frequency (n)	Mean	SD	t/ F value	p-value
**Gender**	Male	61	3.9	0.88	−0.91	0.63
	Female	153	4.0	0.44		
**Place of residence**	City	94	4.0	0.44	0.19	0.82
	Village	115	3.9	0.70		
	Camp	5	3.8	0.59		
**High school stream (Tawjihi)**	Scientific	162	3.9	0.58	−1.10	0.62
	Literary	52	4.0	0.66		
**High school average**	70–79.9	135	3.9	0.69	0.62	0.76
	80–89.9	65	4.0	0.40		
	90–99	14	3.9	0.39		
**Current major**	Nursing	121	4.0	0.72	1.40	0.14
	Non-nursing health profession	93	3.9	0.37		
**Hospital admission or accompaniment experience**	Yes	190	4.0	0.58	1.90	0.04*
	No	24	3.7	0.71		
**Having a family member who is a nurse or nursing student**	Yes	72	3.9	0.75	−0.88	0.38
	No	142	4.0	0.51		

*Note. SD* = Standard Deviation. *p* < .05 indicates statistical significance.

Table 4 shows that gender, age, GPA, and career preference significantly predict total NIS scores. Males, older students, higher GPA, and those preferring nursing as a career reported higher scores. The model explained 32% of variance (Adjusted R^2^ = 0.29), indicating a moderately strong predictive capacity.

**Table 4:** Linear regression analysis for predictors of total Nursing Image Scale (NIS) scores (N = 214).

Predictor variable	B (Unstandardized)	SE	β (Standardized)	t	p-value	95% CI for B
Gender (Male = 1, Female = 0)	2.15	0.74	0.18	2.91	0.004	0.69–3.61
Age	0.12	0.05	0.16	2.40	0.017	0.02–0.22
GPA	1.36	0.52	0.14	2.62	0.009	0.34–2.38
Career preference (Yes = 1)	3.42	1.01	0.21	3.38	0.001	1.43–5.41
Constant	10.42	2.11	—	4.94	<0.001	6.28–14.56

*Model fit*: R^2^ = 0.32; Adjusted R^2^ = 0.29; F(4, n−5) = 15.6; *p* < 0.001.

*Note. B* = Unstandardized coefficient; *SE* = Standard error; *β* = Standardized coefficient; *CI* = Confidence interval.

To strengthen the analytical rigor, we employed multivariable linear regression to examine the association between the Nursing Image Scale (NIS) score (dependent variable) and key sociodemographic and experiential predictors (gender, age, high school stream, high school average, current major, prior hospital exposure, and having a family member in nursing). This approach allowed simultaneous adjustment for potential confounding variables. We verified model assumptions by evaluating normality of residuals, homoscedasticity, and multicollinearity (Variance Inflation Factor < 5 was considered acceptable). Results were reported as unstandardized β coefficients with 95% confidence intervals (CI) and p-values. In addition, Analysis of Covariance (ANCOVA) was conducted to compare adjusted mean NIS scores across major groups (nursing vs. non-nursing) and hospital exposure categories, controlling for covariates. We tested for interaction effects (Major × Hospital Exposure) and calculated effect sizes (Hedges’ g for pairwise comparisons and partial η² for ANCOVA). All analyses were performed using IBM SPSS Statistics version 29, and a two-tailed p-value < 0.05 was considered statistically significant.

Table 5 demonstrates that gender, age, GPA, current nursing major, and prior hospital exposure significantly predicted higher NIS scores. High school stream and family member in nursing were not significant. The model explained 32% of score variance (Adjusted R^2^ = 0.29), indicating a meaningful predictive relationship overall.

**Table 5:** Multivariable linear regression predicting total Nursing Image Scale (NIS) scores (N = 214).

Predictor	B (Unstandardized)	95% CI for B	p-value
Gender (Male = 1, Female = 0)	2.15	0.69–3.61	0.004
Age	0.12	0.02–0.22	0.017
High school GPA	1.36	0.34–2.38	0.009
Current major (Nursing = 1)	3.42	1.43–5.41	0.001
Prior hospital exposure (Yes = 1)	1.87	0.23–3.51	0.025
High school stream	0.74	−0.85–2.33	0.360
Family member in nursing (Yes = 1)	0.92	−0.74–2.58	0.275
Constant	10.42	6.28–14.56	<0.001

*Model summary*: R^2^ = 0.32; Adjusted R^2^ = 0.29; F(7, 206) = 13.6; *p* < 0.001.

*Note. B* = Unstandardized coefficient; *CI* = Confidence interval.

### NCOVA and adjusted group means

An ANCOVA controlling for gender, age, high school stream, GPA, and family member in nursing revealed significant differences in adjusted NIS scores between groups. Nursing students had a higher adjusted mean score (M = 81.2, SE = 1.2) compared to non-nursing students (M = 77.5, SE = 1.4), F(1, 206) = 12.4, p < 0.001, partial η² = 0.057 (a small-to-moderate effect size).

Students with prior hospital exposure scored higher (M = 80.9, SE = 1.0) than those without (M = 76.3, SE = 1.8), F(1, 206) = 9.2, p = 0.003, partial η² = 0.043.

The Major × Hospital Exposure interaction was not significant (p = 0.176), indicating that the effect of exposure was consistent across majors.

Effect Sizes: Pairwise comparisons yielded Hedges’ g = 0.43 for major differences and Hedges’ g = 0.37 for hospital exposure differences, both indicating small-to-moderate practical significance.

**Fig 1:**
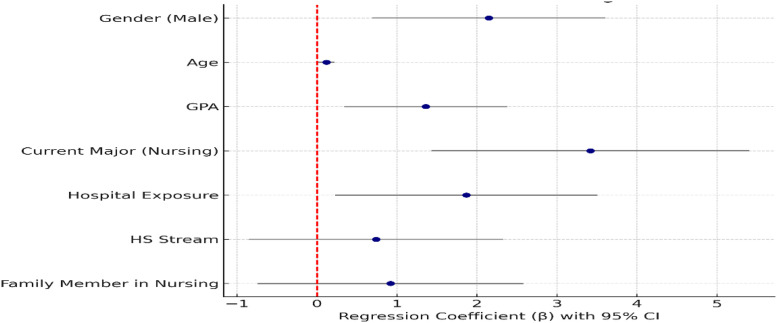
Forest plot of multivariable regression coefficient.

Fig 1 illustrates the adjusted effects of sociodemographic and experiential factors on Nursing Image Scale scores. Positive coefficients indicate higher perception scores. Current major (nursing), gender (male), prior hospital exposure, and GPA significantly predicted more favorable nursing images, while high school stream and family ties showed non-significant effects after adjustment.

**Fig 2:**
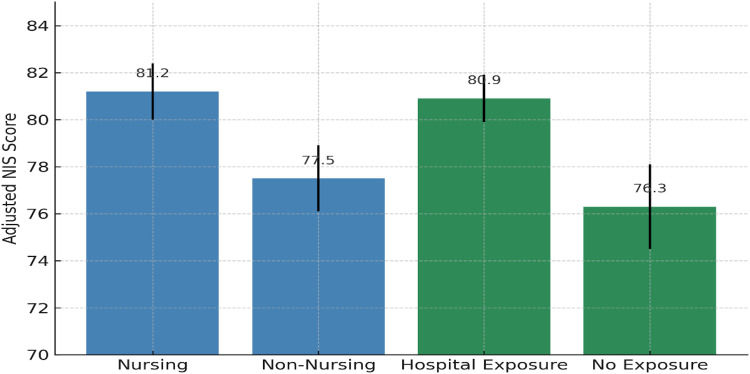
Adjusted means by major and hospital exposure.

As in Fig 2 shows that students majoring in nursing (M = 81.2) had higher adjusted NIS scores compared with non-nursing majors (M = 77.5). Similarly, students with prior hospital exposure (M = 80.9) scored higher than those without exposure (M = 76.3), highlighting the positive influence of academic major and clinical experience.

## Discussion

This study explored how first-year health profession students perceive the nursing profession at Palestine Polytechnic University. The overall finding of moderately positive perceptions (79.8%) indicates a neutral-to-positive stance toward nursing, with slightly higher scores among nursing students compared to peers from other health disciplines. Importantly, prior hospital exposure and stronger academic achievement emerged as consistent predictors of more favorable views. These results highlight that both experiential and individual factors shape early professional perceptions.

Our findings align with international literature showing that perceptions of nursing are influenced by cultural, social, and educational contexts [6,7,19]. In countries such as Turkey and the United States, nursing is increasingly viewed as a respected profession [5,12,20], whereas in Jordan, Saudi Arabia, and parts of sub-Saharan Africa, it is often considered a lower-status career compared to medicine or pharmacy [2,8,21]. This contrast suggests that while Palestinian students hold moderately positive views, they are not yet strongly favorable, reflecting a need for targeted interventions to enhance the professional perception of nursing in the local context.

Hospital exposure emerged as a particularly strong factor associated with positive perceptions. Consistent with previous Palestinian and regional studies, clinical experiences help students observe the autonomy, skills, and leadership of nurses, countering stereotypes that depict nurses as subordinate to physicians or limited to basic care [20,21]. This underscores the importance of structured early exposure for all health profession students, not only those enrolled in nursing. Contact theory further explains these results: direct interaction with nurses in authentic clinical environments reduces misconceptions and promotes a more accurate understanding of their professional role [12].

Psychological resources such as resilience and academic achievement were also associated with perceptions that are more positive. Earlier Palestinian research demonstrates that resilience and psychological well-being protect students against stress during training, particularly in contexts of political instability and violence [22,23]. Our results suggest that resilient and high-achieving students may be better equipped to appreciate the intellectual and humanitarian dimensions of nursing. These findings support integrating resilience-building strategies and emotional intelligence training into health curricula to reinforce professional identity formation [24].

The political and social context of Palestine adds a unique dimension. Despite instability and conflict, students still reported moderately positive perceptions, reflecting recognition of nursing as a socially valuable and humanitarian profession. This reinforces the notion that nursing is not merely seen as a career choice but also as a vocation serving communities in crisis [19,25]. Such findings position Palestinian nursing education within broader international discussions on how sociopolitical environments influence professional identity [10,11].

From a theoretical perspective, the results are consistent with Social Cognitive Career Theory, which emphasizes the role of experiential learning in shaping outcome expectations. Students exposed to clinical environments developed more favorable perceptions, while academic success contributed to self-efficacy and professional pride. These mechanisms highlight the importance of both environmental opportunities and personal resources in career-related decision-making [13,14,26].

**Practical implications** are clear. The most feasible strategies in the Palestinian context include: (1) integrating structured early clinical exposure across all health programs, (2) incorporating interprofessional education where nursing students take leadership roles, and (3) embedding resilience-building and emotional intelligence training in nursing curricula. Public campaigns should also highlight male nurses and advanced practice roles to challenge persistent gender stereotypes. These interventions align with WHO and ICN recommendations to strengthen the percipation of nursing as part of addressing workforce shortages [10,11,26,27].

**Strengths and limitations** must be acknowledged. This is the first study in Palestine to investigate perceptions of nursing among first-year students using a culturally adapted scale. However, its single-institution setting, cross-sectional design, convenience sampling, and reliance on self-reported data limit generalizability. Longitudinal and multi-site studies, supplemented with qualitative research, are recommended to provide a deeper understanding of students’ evolving professional perceptions.

## Conclusion

This study provides novel evidence on the perceptions of nursing among first-year health profession students in Palestine, highlighting the role of prior hospital exposure, resilience, and academic achievement in shaping nursing’s professional image. While the overall perception was moderately positive, significant variations indicate that experiential and psychological factors critically influence attitudes toward the profession. By integrating these findings with contemporary literature on stress, resilience, and emotional intelligence this research contributes to the scientific understanding of how early exposure and personal resources interact to form professional identity in nursing.

From a scientific perspective, the study advances knowledge in three ways. First, it empirically demonstrates that early, structured exposure to healthcare settings is a determinant of positive perceptions of nursing, confirming theoretical models such as Social Cognitive Career Theory in a Middle Eastern context. Second, it expands the growing body of literature linking resilience and emotional competencies to career image, suggesting that psychological resources are as important as educational design in influencing professional choice. Third, it situates the discussion within a politically unstable environment, thereby providing unique insights into how nursing is perceived under conditions of chronic stress and conflict.

Together, these contributions underscore that strengthening the image of nursing is not only a matter of correcting stereotypes but also of building resilient, emotionally intelligent, and academically supported students. The findings therefore add to international scientific discourse by offering context-specific evidence from Palestine, a setting where nursing’s societal role is both urgent and under-researched.

## Supporting information

S1 File The questionnaire assesses nursing image perception among first-year nursing and health science students. It includes demographic data and a 26-item Nursing Image Scale covering appearance, communication, and professional roles.
(DOCX)
