# Supplementary material for: Perceptions of the nursing profession among first-year health profession students: A cross-sectional study in Palestine
Source: PLoS One. 2025 Oct 24;20(10):e0334933. doi: 10.1371/journal.pone.0334933 (PMC12551881; doi:10.1371/journal.pone.0334933)
Supplement: S1 File — It includes demographic data and a 26-item Nursing Image Scale covering appearance, communication, and professional roles. (DOCX) [file pone.0334933.s001.docx]

**College of Nursing**

**Scientific research Questionnaire**

**Greetings, and after due salutation…**

The research team is in the process of formulating a study regarding

**"The Perception of the Nursing Profession among First-Year Nursing Students and Other Health Science Students at Palestine Polytechnic University.”**

You have been selected as part of the study sample. Therefore, we present to you this questionnaire as an integral component of the study to attain a Bachelor's degree in Nursing from Palestine Polytechnic University - Hebron. We kindly seek your assistance by responding to the survey questions accurately and objectively, recognizing its significant impact on achieving precise results. It is imperative to note that all provided information will be held in strict confidentiality, solely for the purpose of scientific research. Participation in the study is entirely voluntary, and there is no need to disclose your esteemed name or any information indicative of your person.

Furthermore, the research team is prepared to furnish you with the study results upon completion, should you wish. We extend our sincere gratitude for your cooperation.

For inquiries, please contact “0598812936”

**Research Team: Fatima Taqatqa, Bayan Deriyh, Ibraheem Thawabteh, Naseem Khlayleh**

**Supervised by Kareem Sbeih (RN, MSN)**

## Appendix (2) : Questionnaire

| **Part one: Socio-demographic data** | |
| --- | --- |
| 1. **Age______** | |
| 1. **Gender** | **□** Male □ Female |
| 1. **School Types** | □ Public □ Private |
| 1. **Marital Status** | □Single  □ Married  □ Others |
| 1. **Residence مكان السكن** | □ City  □ Village  □ Camp |
| 1. **Tawjehi Specialization ة** | □Scientific  □Literary |
| 1. **Tawjehi. Average** 2. **College** | □90-99  □80-89  □70-79  □ Nursing ا  □ Medical and health sciences |
| 1. **Income** | □less than أقل من1880 □ (1880-2500)  □ More than أكثر من 2500 |
| 1. **Has a relative or friend who works as a nurse?** | □Yes □No |
| 1. **Family reactions when talking about enrollment in nursing faculty** | □Positive  □Negative  □Neutral |
| 1. **Having knowledge of the profession** | □ Yes □ No |
| 1. **If yes, what is the source of your knowledge?** | □Social Medi  □Relatives  □Television  □Media  □Friends  □School  □Other________ |

**Second Part: Nursing Image Perception scale**

| **Nursing Image perception** | Strongly disagree | Disagree | Neutral | Agree | Strongly Agree |
| --- | --- | --- | --- | --- | --- |
| **A. General appearance** | | | | | |
| A.1 Nurses’, who are well-maintained and clean persons. |  |  |  |  |  |
| A.2 Nurses’ who are polite and respectful persons. |  |  |  |  |  |
| A.3 Nurses’ who are cheerful and friendly persons. |  |  |  |  |  |
| A.4 Nurses’, who are authoritarian and tough-looking person. |  |  |  |  |  |
| A.5Nurses’, who are uniformed persons. |  |  |  |  |  |
| **B. Communication** | | | | | |
| B.1 Nurses’, listens people. |  |  |  |  |  |
| B.2 Nurses’, allows people to ask questions. |  |  |  |  |  |
| B.3 Nurses’ provide solutions to the questions of healthy individuals |  |  |  |  |  |
| B.4 Nurses’ provide solutions to the questions of the patient. |  |  |  |  |  |
| B.5 Nurses’ are keeping secrets.  . |  |  |  |  |  |
| B.6 Nurses’ are the guidance consultants. |  |  |  |  |  |
| **Occupational and educational characteristics** | | | | | |
| C.1 Nurses’ working conditions are difficult. |  |  |  |  |  |
| C.2 Nurses can work in many institutions outside the hospital (factories, schools).). |  |  |  |  |  |
| C.3 Nursing education should be at the university level. |  |  |  |  |  |
| C.4 Nurses can be administrator. |  |  |  |  |  |
| C.5 Nurses can be teachers. |  |  |  |  |  |
| C.6 Nurses can carry out research |  |  |  |  |  |
| C.7 Nurses can be associate professors and professors.  . |  |  |  |  |  |
| C.8 Nursing is a profession that independent practices |  |  |  |  |  |
| C.9 Nursing care has vital importance in the recovery of patients. |  |  |  |  |  |
| C.10 Nursing is a profession based on knowledge |  |  |  |  |  |
| C.11 Nursing is a profession based on skills. |  |  |  |  |  |
| C.12 In nursing services, individual and community health education is important. |  |  |  |  |  |
| C.13 Males can be nurses. |  |  |  |  |  |
| C.14 Nurses are patient advocates.  ا |  |  |  |  |  |
| C.15 Nurses’ has a high prestige in the community. |  |  |  |  |  |
